# EB3 Regulates Microtubule Dynamics at the Cell Cortex and Is Required for Myoblast Elongation and Fusion

**DOI:** 10.1016/j.cub.2007.06.058

**Published:** 2007-08-07

**Authors:** Anne Straube, Andreas Merdes

**Affiliations:** 1 Wellcome Trust Centre for Cell Biology, University of Edinburgh, King's Buildings, Edinburgh EH9 3JR, Scotland, United Kingdom; 2 Cytoskeletal Organization Laboratory, Marie Curie Research Institute, Trevereux Hill, Oxted, Surrey RH8 0TL, United Kingdom

**Keywords:** CELLBIO, DEVBIO

## Abstract

During muscle differentiation, myoblasts elongate and fuse into syncytial myotubes [1]. An early event during this process is the remodeling of the microtubule cytoskeleton, involving disassembly of the centrosome and, crucially, the alignment of microtubules into a parallel array along the long axis of the cell [2–5]. To further our understanding on how microtubules support myogenic differentiation, we analyzed the role of EB1-related microtubule-plus-end-binding proteins. We demonstrate that EB3 [6] is specifically upregulated upon myogenic differentiation and that knockdown of EB3, but not that of EB1, prevents myoblast elongation and fusion into myotubes. EB3-depleted cells show disorganized microtubules and fail to stabilize polarized membrane protrusions. Using live-cell imaging, we show that EB3 is necessary for the regulation of microtubule dynamics and microtubule capture at the cell cortex. Expression of EB1/EB3 chimeras on an EB3-depletion background revealed that myoblast fusion depends on two specific amino acids in the calponin-like domain of EB3, whereas the interaction sites with Clip-170 and CLASPs are dispensable. Our results suggest that EB3-mediated microtubule regulation at the cell cortex is a crucial step during myogenic differentiation and might be a general mechanism in polarized cell elongation.

## Results and Discussion

### EB Family Members Are Transcriptionally Regulated during Myogenic Differentiation

**Figure 1:**
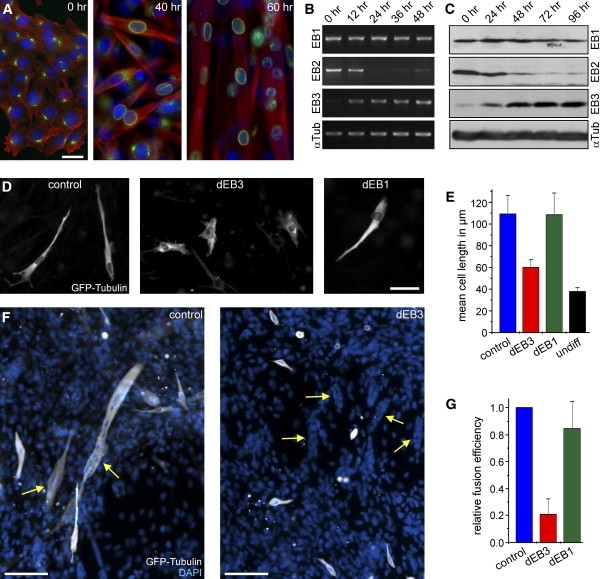
EB Proteins during Myogenic Differentiation (A) Morphological changes and reorganization of the microtubule cytoskeleton. Immunofluorescence of C2C12 cells that were shifted to differentiation for the times indicated and stained for α-tubulin (red), PCM-1 (green), and DAPI (blue) is shown. The scale bar represents 20 μm. (B) Reverse-transcription polymerase chain reaction (RT-PCR) of EB1, EB2, EB3, and α-tubulin from RNA isolated from C2C12 myoblasts that were cultured under differentiation conditions for the times indicated. (C) Western blots of total cell lysates of C2C12 cells that were cultured under differentiation conditions for the times indicated and probed with antibodies against EB1, EB2, EB3, and α-tubulin. (D) C2C12 myoblasts, cultured under differentiation conditions for 41 hr, 3 days after transfection with shRNA constructs against luciferase (control), EB3, and EB1, coexpressing GFP-tubulin. The scale bar represents 50 μm. (E) Average of the mean cell length in four to seven cell-elongation experiments after 19–54 hr under differentiation conditions. The average cell size of undifferentiated myoblasts is included for comparison. Error bars represent the standard deviation (SD). (F) Myoblast fusion after 54 hr differentiation. GFP-tubulin (white) highlights cells that were transfected with the shRNA constructs (control and EB3); DNA staining is shown in blue. Clusters of nuclei (arrows) indicate myoblast fusion in both fields of view. Note that cells transfected with EB3 shRNAs do not participate in fusion. The scale bar represents 100 μm. (G) Quantification of myoblast-fusion efficiency after 49–54 hr under differentiation conditions. Because of variations between experiments, the efficiency of cell fusion (percentage of cells containing two or more nuclei) was set to 100% for control cells in each experiment. Columns represent the average of three to four experiments, and error bars represent the SD.

Microtubule integrity is essential for muscle cell differentiation [7–9]. To further our understanding of how the microtubule cytoskeleton supports myoblast elongation and fusion, we induced the murine myoblast cell line C2C12 to differentiate in vitro (see Supplemental Experimental Procedures in the Supplemental Data available online). Within one day, myoblasts elongated to become spindle shaped, the microtubule cytoskeleton was rearranged into a parallel array [4], and centrosome proteins relocalized to the nuclear periphery (Figure 1A, Figure S1A). Subsequently, differentiating myoblasts fused preferentially at the cell tips to form multinucleated myotubes [10] (Figure 1A). Because the cell tips are characterized by a high density of microtubule plus ends (data not shown), we studied the potential role of microtubule tip-binding proteins of the EB family. Mammalian cells possess three family members—EB1, EB2, and EB3 [11]—that share a conserved domain structure and show high sequence similarity (Figure S1B). However, we found that murine EB proteins were differentially expressed during myogenesis (Figures 1B and 1C): Both EB1 mRNA and protein levels remained constant during differentiation, whereas EB2 expression was rapidly switched off once cells started to differentiate. In contrast, EB3 that was expressed at a low basal level in undifferentiated cells was highly upregulated early during differentiation (Figures 1B and 1C). The expression pattern of EB1 and EB3 during myogenic differentiation was confirmed with primary myoblasts from the mouse *H-2K*^b^-tsA58 [12] (Figure S1C). As in other cell types, EB3 was found to localize to microtubule plus ends in myoblasts and myotubes (Figure S1A). Its expression pattern suggested that EB3 might play an important role in the reorganization of the microtubule cytoskeleton during myogenic differentiation. Because EB2 was shut down immediately, it was not included in the functional analysis below.

### Depletion of EB3 Affects Microtubule Organization in  Myoblasts

In order to study the function of EB proteins, we used vector-based RNA-interference constructs that allowed the simultaneous expression of an RNA hairpin and green fluorescent protein (GFP) as a marker protein to identify successfully transfected cells [13]. Target sequences were chosen to specifically deplete EB1 and EB3 separately, as well as both proteins simultaneously. A small hairpin RNA (shRNA) construct targeting firefly luciferase was used as a control. The depletion efficiency was 90% or higher for all constructs used (Figure S2). Different from COS-7 and HeLa S3 cells [14, 15], EB1 depletion in undifferentiated myoblasts seemed to have no negative effect on the centrosomal focusing of microtubule minus ends, and microtubule organization appeared to be normal (Figure S3). However, the depletion of EB3 affected microtubule organization in undifferentiated cells, although EB3 was only expressed at low levels (Figures 1B and 1C). Microtubule distribution was uneven, with some areas of the cytoplasm containing only very few microtubules and other areas being crowded with curled microtubules (Figure S3). This phenotype of curled microtubules was even more pronounced in cells depleted of both EB1 and EB3 (Figure S3), suggesting that EB1 shares partly redundant functions with EB3. This is in concordance with previous reports that EB1 and EB3 have common interacting partners, such as adenomatosis polyposis coli (APC), Dynactin, Clip-170, Clasp1, and Clasp2 [16–18], and that both proteins are redundant in regulating microtubule dynamics [17]. Moreover, EB3 localization at microtubule tips was increased after the depletion of EB1 in C2C12 cells (Figure S2), further supporting the notion that EB3 can compensate for the loss of EB1.

### Depletion of EB3 Impairs Myoblast Elongation and Fusion

Because we found that EB3 protein expression increased once myoblasts started to differentiate, we wanted to test whether depletion of EB3 would interfere with any aspect of differentiation. For this purpose, differentiation was induced at least 20 hr after transfection with shRNA, and cells were analyzed 19 to 54 hr later. At this time point, most control cells were spindle shaped and had a mean length of about 110 μm (109.5 ± 17.2 μm; Figures 1D and 1E). The depletion of EB1 did not interfere with cell elongation (Figure 1D): The cell-length distribution (data not shown) and the mean cell length in EB1-depleted cells (108.9 ± 19.9 μm) were very similar to those of control cells (Figure 1E). In contrast, depletion of EB3 resulted in significantly shorter cells (59.7 ± 7.1 μm; Figures 1D and 1E). For comparison, undifferentiated C2C12 cells have a mean length of 37.6 ± 3.8 μm. Thus, EB3-depleted cells manage to increase their cell length upon differentiation by less than 60% of their original size, whereas control cells elongate to about 3-fold length. Similar data were obtained regardless of whether GFP or a GFP-tubulin fusion protein were used as reporter constructs.

In order to analyze the effect of EB1 or EB3 depletion on myoblast fusion, cells were transfected with shRNA constructs coexpressing GFP-tubulin, shifted to differentiate after 20–24 hr, and analyzed 50–54 hr later. Although frequent fusion into syncytial myotubes could be observed in transfected control cells (Figure 1F), the depletion of EB3 drastically reduced the number of fused myoblasts (Figures 1F and 1G). The small proportion of fused cells that were found among the GFP-tubulin-expressing cells were undistinguishable from control cells and usually not effectively depleted of EB3 (data not shown). This implies that the complete absence of EB3 might not permit myoblast fusion at all. Although clusters of nuclei indicated syncytial myotubes in the field of view, cells expressing EB3 shRNAs did not participate in cell fusion (Figure 1F), suggesting that depletion of EB3 had a dominant effect, probably by interfering with the acquisition of fusion competence. Although the depletion of EB1 had no effect on cell elongation (see above), it had minor effects on myoblast fusion and reduced the number of fused myoblasts to about 80% of control values (Figure 1G). Because the expression of EB1 hairpin constructs increased the frequency of apoptosis (not shown), the effect on myoblast-fusion efficiency might be caused solely by the reduction of cell density. In the double-depletion experiment, only few transfected cells survived (data not shown), suggesting that the absence of both EB1 and EB3 during differentiation is lethal.

### Expression of Myogenic Markers Is Not Affected after EB3 Depletion

**Figure 2:**
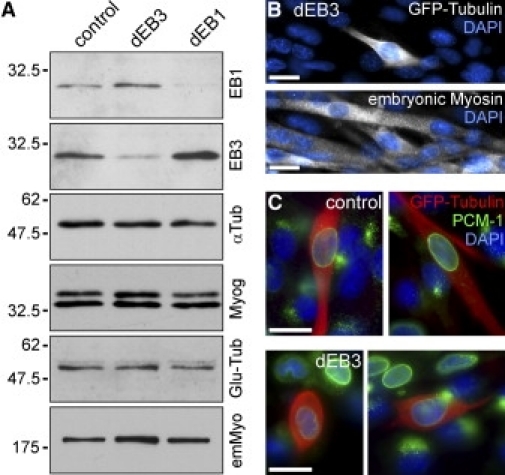
Expression of Myogenic Markers Is Not Affected after EB3 Depletion (A) Western-blot analysis of 20,000 GFP-expressing cells after fluorescence-activated cell sorting (FACS), 3 days after transfection with shRNA constructs. Blots were probed with antibodies against EB1, EB3, α-tubulin, myogenin, Glu-tubulin, and embryonic myosin. Positions of molecular weight markers are indicated on the left. (B) Immunofluorescence staining for embryonic myosin in EB3-depletion experiments after 55 hr in differentiation conditions. In the top panel, GFP-tubulin (white) highlights a transfected cell; in the bottom panel, embryonic myosin staining (white) of the same group of cells is shown. DNA is shown in blue. Scale bars represent 20 μm. (C) Immunolocalization of PCM-1 (green) after 55 hr in differentiation conditions. GFP-tubulin (red) highlights transfected cells with either control (upper row) or EB3 shRNAs (lower row). DNA is shown in blue. Scale bars represent 20 μm.

To further investigate the molecular mechanisms of EB3-dependent myoblast fusion, we tested whether EB3 depletion interferes with the timely regulated expression of transcription factors and structural proteins that are specific for myogenic differentiation. The expression level of the muscle-specific transcription factor myogenin was not notably affected by depletion of EB1 or EB3 (Figure 2A). Likewise, the depletion of EB1 or EB3 had no negative effect on the expression of embryonic myosin, which is a structural component of the contractile apparatus and exclusively expressed during myogenesis (Figures 2A and 2B). Another early event of myoblast differentiation is the relocation of centrosomal proteins to the nuclear surface. We found no significant difference in the efficiency with which the centrosome protein PCM-1 was relocated between control and EB3-depleted cells (Figure 2C). This suggests that the reorganization of microtubule minus ends is initiated correctly. Further, we determined that the stabilization of microtubules during myogenic differentiation, as indicated by the accumulation of posttranslational tubulin modifications [19], occurs normally: The levels of acetylated (data not shown) and detyrosinated (Glu-) tubulin were found to be unaltered in EB1- or EB3-depleted cells (Figure 2A). Taken together, these observations suggest that myoblasts that were depleted of EB3 progress through the early steps of myogenesis, and that their failure to elongate and fuse might be a direct effect of the limited supply of EB3.

### EB3-Depleted Myoblasts Show Defects in Cell Spreading, Elongation, and Fusion Independently of the Adhesion to the Extracellular Matrix

Because problems with cell elongation could be caused by defects in attachment to the extracellular matrix, we compared the efficiency of a variety of adhesion molecules and artificial substrates to affect cell spreading in an EB3-depletion background. Most control cells were spread on polylysine, fibronectin, or collagen after 70 min (Figure S4A2). Although attachment to the substrates was comparable in control and EB3-depleted cells, significant spreading was observed only in less than 50% of EB3-depleted cells, on each substrate provided (Figure S4A). Even those EB3-depleted cells that spread showed differences from control cells because they occupied a smaller surface area (Figures S4B and S4C). Furthermore, providing different surface coatings did not rescue myoblast elongation or fusion efficiency (Figures S4D and S4E). These data suggest that EB3 depletion does not affect any particular transmembrane complex for cell adhesion, but instead affects the microtubule-based support of cell spreading and elongation.

### Microtubule Behavior at the Cell Cortex Is Altered in EB3-Depleted Cells

**Figure 3:**
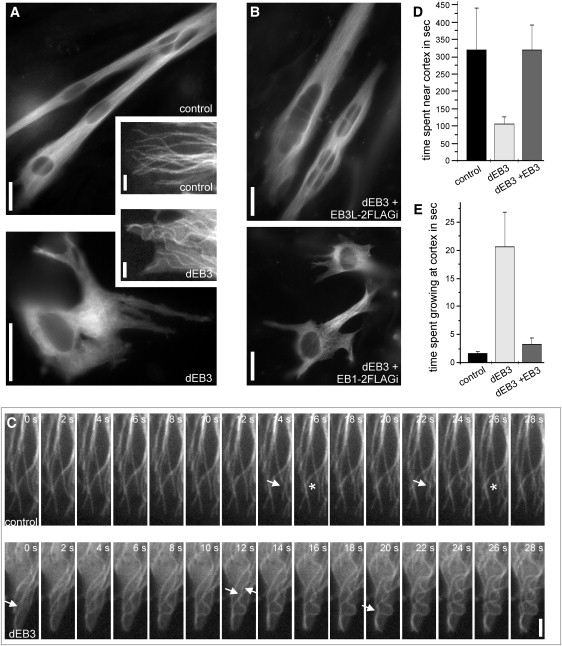
Microtubule Behavior at the Cell Cortex is Altered in EB3-Depleted Cells (A) Microtubule organization in C2C12 cells 3 days after transfection with shRNA constructs and after 52 hr under differentiation conditions. GFP-tubulin highlights the microtubule cytoskeleton in transfected cells. The insets show the cell edges of differentiating myoblasts after 49 hr under differentiation conditions. Scale bars represent 20 μm, and 2 μm in the insets. (B) Microtubule organization in C2C12 cells 3 days after cotransfection of the EB3 shRNA construct and rescue constructs for full-length EB3 or EB1. GFP-tubulin highlights the microtubule cytoskeleton in transfected cells. Scale bars represent 20 μm. (C) Images taken from time-lapse movies of GFP-tubulin in shRNA transfected cells that were shifted to differentiation for 49 hr. The time in seconds is indicated in the upper-right corner. Arrows highlight newly appearing bumps in microtubules that reach the cell cortex and continue growth. Asterisks indicate the relaxation of such bumps. The scale bar represents 2 μm. See the Supplemental Data for the respective movies. (D) Quantification of the time microtubule tips spent at or close to the cortex (maximum of 2 μm away). Columns represent data obtained from 93–129 microtubules in five to eight cells, and error bars indicate SD. Microtubule tips spend significantly less time close to the cortex in EB3-depleted compared to control cells (analysis of variance [ANOVA]: p = 0.0003). (E) Quantification of the time microtubules continue to grow after reaching the cell cortex, resulting in the introduction of bends into microtubules. Columns represent data obtained from 38–64 microtubules in four to eight cells, and error bars indicate SD.

**Figure 4:**
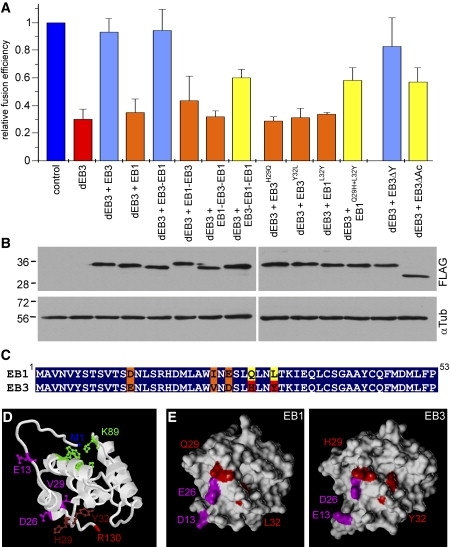
Rescue of EB3 Depletion (A) Quantification of myoblast fusion efficiency after 54–55 hr under differentiation conditions. The efficiency of cell fusion (percentage cells containing two or more nuclei) was set to 1.0 for control cells in each experiment (ranging from 8% to 34% fused cells), and relative values are shown for all other constructs. Columns represent an average of three to four experiments, and error bars indicate the SD. The columns representing values not significantly different from those of control cells (ANOVA: p > 0.05) are shown in light blue, whereas values not significantly different from those of EB3 deletion are shown in orange (ANOVA: p > 0.05). A partial rescue (which falls in neither of these categories, with p < 0.02) is shown in yellow. (B) Western blots of extracts of GFP-positive cells (after FACS sorting) expressing the rescue constructs indicated in Figure S5, as well as EB3 shRNAs. Blots were probed with antibodies against FLAG and α-tubulin. (C) Sequence alignment showing the first 53 amino acids of murine EB1 and EB3 (Swiss-Prot accession numbers Q61166 and Q6PER3, respectively). Identical amino acids are highlighted in blue, similar amino acids in orange, and sequence differences in yellow and red. (D) Representation of the amino acid changes that confer partial rescue to the EB1 construct in the 3D structure of EB3 N-terminal domain (PDB accession number 1WYO, model 1, amino acids 8–137). The putative microtubule-binding site around K89 is shown in green [22], and the five crucial amino acid changes between EB1 and EB3 are shown in magenta or dark red. The image was created with Protein Explorer 2.78 (http://www.proteinexplorer.org). (E) Surface maps of N-terminal domains (amino acids 1–130) of EB1 and EB3 were created with SYBYL 6.9.1 on the basis of PDB files 1PA7 and 1WYO. The four surface-exposed amino acid changes are highlighted in pink and red. Note that the surface structure changes as Y32 in EB3 fills a deep cleft present in EB1, and amino acids H29 and D26 emanate from the surface of the molecule.

In order to study the influence of EB3 depletion on the microtubule cytoskeleton, we monitored the behavior of microtubules marked with GFP-α-tubulin in fixed and live cells. Differentiated control cells showed a dense microtubule array oriented parallel to the longitudinal axis of the cell (Figure 3A). EB3-depleted cells often showed aberrant cell shapes with several short extensions in various directions but were missing a clear axis. Furthermore, microtubules were disorganized and curled, a phenotype that was already visible in undifferentiated cells, although less pronounced (Figure 3A, Figure S3). Defects in cell shape and microtubule organization in EB3-depleted cells were rescued by coexpression of EB3 with an internal double FLAG tag (Figures 3B and 4B, Figure S5). Equivalent experiments with the respective EB1 construct failed to rescue (Figure 3B).

Whereas microtubule plus ends extended straight into the cell cortex in control cells, the formation of curls was frequently observed in EB3-depleted cells (insets in Figure 3A). Live-cell imaging of control depleted cells revealed that most microtubules reaching the cortex immediately stopped growing and switched to a dynamic “captured” state with very short, alternating phases of growth and shrinkage that kept the microtubule plus ends close to the cell cortex for an extended period of time (Figures 3C and 3D, Movie S1). The capturing of microtubules involves the stabilization by rescue factors (such as CLASPs) that lower the frequency of long depolymerizing phases and increase microtubule longevity [20]. In addition, microtubule destabilizers (such as stathmin or kinesin-13 family members) are thought to trigger depolymerization from microtubule ends that reach the cell cortex [21] and thus balance the action of rescue factors to maintain a constant microtubule length. Live-cell imaging of GFP-labeled microtubules in EB3-depleted cells revealed defects in both aspects of cortical microtubule capture: The dwell time of microtubule ends in the cortical area was found to be reduced 3-fold compared to that of control cells (Figure 3D, Table 1). In addition, most microtubules continued growing after reaching the cell borders, resulting in the bending of the polymer behind the tip (Figures 3C and 3E, Movie S2). Such behavior was observed on 90% of microtubules in the EB3 knockdown, with microtubules spending on average 20.3 s in the growth phase after reaching the cortex. In comparison, only about 20% of microtubules in control cells continued growing for a short period (7.8 s on average), resulting in an average time of 1.7 s per microtubule observed (Figure 3E). Expression of EB3-2FLAGi rescued both phenotypes in cortical microtubule behavior (Table 1). The average dwell time of microtubule tips at the cell cortex was 5.3 min, which is almost identical to control values (Figure 3D). Although 58% of microtubules still showed buckling after hitting the cortex, they did so for a short period only (6.2 s on average), resulting in an average time of 3.3 ± 0.99 s per microtubule observed (Figure 3E). This was not significantly different from that of control cells (analysis of variance (ANOVA): p = 0.098). These data demonstrate that EB3 is required for the regulation of microtubule dynamics in a way that allows microtubule capture at the cell edge.

In order to test whether other parameters of microtubule dynamic instability were affected, we measured growth and shrinkage velocities in control and EB3-depleted cells. Although microtubules grew continuously for shorter periods only in the absence of EB3 (data not shown), microtubule growth speeds were comparable to those of control cells (Table 1). However, shrinkage speeds were significantly reduced after EB3 depletion (Table 1). It has to be noted that microtubule catastrophe events occur at a higher speed when they originate from the cell cortex than do events induced in the cytoplasm (cortical: 0.600 ± 0.156; cytoplasmic: 0.431 ± 0.136, n = 19–28 in 3 cells). The cytoplasmic depolymerization speed in control cells resembles that of cortical depolymerizations in EB3-depleted cells, further supporting the notion that cortical regulation of microtubule dynamics is dependent on EB3. As shown for other microtubule phenotypes, shrinkage speeds were rescued by the expression of a full-length EB3 construct (Table 1).

### The N-Terminal Domain of EB3 Is Necessary for Its Myogenic Function

Because both EB1 and EB3 share high sequence homology, we went on to test which domains of EB3 were conferring myoblast-specific microtubule behavior and myoblast fusion, by rescuing EB3-depleted cells with a variety of constructs encoding EB3, EB1, and chimaeras of the two proteins. Myoblast-fusion efficiency was rescued almost completely by coexpressing full-length EB3-2FLAGi with the EB3 shRNA construct (Figure 4A). In addition, elongated cell shape, parallel microtubule organization, and microtubule behavior at the cell cortex was restored (see above; Figure 3, Table 1). Full-length EB1-2FLAGi could not substitute for the loss of EB3, indicating that the observed defects are not caused by the lower amount of total EB proteins present and that EB3 fulfils a specific function in myoblast fusion (Figures 3B and 4A). Next, we constructed EB1-EB3 chimeras by swapping domains between these proteins, to map functionally important EB3-specific regions (Figure S5). The EB3-2FLAG-EB1 construct fully rescued myoblast fusion, whereas EB1-2FLAG-EB3 was ineffective (Figure 4A). This finding was unexpected because the N-terminal half contains the microtubule-binding region and is highly conserved (82% identity, 92% homology within first 142 amino acids of EB1 and EB3). In order to map the crucial region more accurately, we swapped smaller regions within the N-terminal domain. Surprisingly, the construct encoding only the first 53 amino acids of EB3 increased myoblast fusion efficiency significantly (EB3-EB1-2FLAG-EB1, Figure S5, Figure 4A). EB1 and EB3 differ only in 5 amino acids in this region, of which three are conservative exchanges (Figure 4C). In order to test whether the exchanges at positions 29 (Q > H), and 32 (L > Y) are indeed crucial for the myogenic functions of EB3, we introduced point mutations to the EB3-2FLAGi rescue construct that amend H29 or Y32 to the amino acids present in EB1. Neither EB3^H29Q^ nor EB3^Y32L^ could rescue the myogenic defects in EB3-depleted cells (Figure 4A). Finally, we introduced the reverse mutations to EB1, and although single mutations at Q29H or L32Y were without effect, the double mutation (EB1^Q29H+L32Y^) increased its ability to rescue myoblast fusion significantly (Figure 4A). Because the binding specificity for microtubule plus ends is higher for EB3 than it is for EB1 (Figure S6), and because the crucial region is located in the microtubule-binding domain of EB3, we thought that the differences in microtubule-binding properties could underlie the specialized function of EB3. However, both mutated constructs, EB3^H29Q^-2FLAGi and EB3^Y32L^-2FLAGi, localized as efficiently to the microtubule tips as did unmutated EB3 (Figure S6). Moreover, these results demonstrate that the calponin-like domains of EB3 still fold properly in the presence of the point mutations.

Next, we localized the differing amino acids in the 3D structure of EB3 (Protein Data Bank [PDB] accession number 1WYO). Four of the five amino acids are exposed at the surface of EB3 that is opposite to the proposed microtubule-binding site [22] (Figure 4D). Because the exchanges at positions 26 (E > D), 29 (Q > H), and 32 (L > Y) alter the surface of the molecule (Figure 4E), they could provide an interaction site for a protein that binds specifically to EB3 but not to EB1.

Clip-170 and CLASPs are interacting proteins of EB3 [17, 18] and were reported to be involved in microtubule capture at the cell cortex [18, 23]. In order to determine whether the myogenic function of EB3 involves the interaction with these proteins, we tested whether truncated EB3 lacking the interaction sites would still be able to rescue the myoblast-fusion phenotype. The deletion of the C-terminal tyrosine residue from EB3 prevents binding to Clip-170 [17]. However, the truncated construct EB3ΔY-2FLAGi is still able to rescue the myoblast-fusion defect in EB3-depleted cells (Figure 4A). The deletion of the C-terminal acidic tail impairs the binding of CLASPs to EB1 [18], and presumably to EB3, as well. Construct EB3ΔAc-2FLAGi lacks the C-terminal 23 amino acids and partially rescues myoblast-fusion efficiency in dEB3 cells (Figure 4A). These observations suggest that Clip-170 is not involved in the phenotype described, whereas CLASPs and other interactors at the acidic tail appear to play a minor role. However, we think that EB3 probably has a specific binding partner in its calponin-like domain that mediates its differentiation-specific function.

Finally, we wanted to test whether the calponin-like domain of EB3 is responsible for the changes in microtubule dynamics at the cell cortex. As described above, all microtubule phenotypes were rescued by the coexpression of EB3-2FLAGi with EB3 shRNAs. In contrast, EB1-2FLAGi increased neither the dwell time of microtubule plus ends near the cell cortex nor the shrinkage speeds from the cell cortex (Table 1). However, microtubule “overgrowing” was rescued by the EB1 construct, suggesting that this phenotype is dependent on the level of EB proteins, rather than being specific for EB3. We then used the EB3-2FLAG-EB1 fusion protein that was competent to fully rescue the myoblast-fusion phenotype. EB3-2FLAG-EB1 could fully rescue the dwell time of microtubule ends at the cell cortex, as well as microtubule overgrowing, and microtubule shrinkage rates were partially rescued with this construct (Table 1). These data demonstrate that the calponin-like domain of EB3 is required for cortical microtubule stabilization, as well as for the induction of fast catastrophes from the cell cortex. Efficient myoblast fusion is dependent on the N terminus of EB3, too, supporting the idea that EB3-dependent regulation of microtubule dynamics at the cell cortex is required to provide the cytoskeletal support for cell elongation and cell-to-cell fusion during muscle cell differentiation.

### How Does EB3 Regulate Microtubules at the Cell Cortex, and What Are the Implications for the Differentiating Muscle Cell?

It is difficult to draw a conclusive model about the myogenic function of EB3, although its close homolog EB1 and common interactors of EB1 and EB3 have been implicated in various aspects of microtubule-dynamics regulation. We show that the dwell time of microtubule ends close to the cell cortex is strongly reduced in the absence of EB3. This suggests that microtubule capture and stabilization is impaired in EB3-depleted cells. However, in their entirety, microtubules are stable and long lived, as evidenced by regular levels of tubulin modifications and the presence of long, curled microtubules in EB3-depleted cells. To understand this paradox, one should consider that capture of microtubule ends at the cell cortex is only one of several ways to stabilize microtubules. The upregulation of a muscle-specific isoform of MAP4 early during muscle differentiation [24] suggests that microtubule stabilization along their length could also contribute to microtubule longevity. Moreover, microtubule ends could be stabilized at contact sites with vesicles or organelles in the cytoplasm, even if cortical capture is compromised in EB3-depleted cells. Finally, changes in the microtubule depolymerization rate, which we found reduced to about 70% after EB3 depletion, could contribute to the phenotype. It is thought that depolymerizing kinesins are loaded to growing microtubule plus ends and become activated once the microtubule reaches the cell cortex [21]. In EB3-depleted cells, the loading or activation of microtubule destabilizers might be defective, explaining both the reduced depolymerization rate and the continuous growth of plus ends seen at the cortex.

Although the different phenotypes of microtubule behavior could be caused by separate EB3-dependent pathways, a general mechanism that underlies the changes of microtubule dynamic behavior within cortical areas could be affected here. This may involve Rho family GTPases, such as RhoA, Rac1, and Cdc42, which have been implicated in microtubule stabilization, together with their effector proteins [23, 25–28]. RhoGEF2, an upstream activator of Rho, was shown to travel to the cell cortex on the tips of growing microtubules by interaction with EB1 [29]. Thus a possible mechanism by which EB3 regulates cortical-microtubule behavior could be the targeting of a specific RhoGEF or similar signaling molecule to the cell cortex. Because Rho-like GTPases are key regulators of the actin cytoskeleton and associated integrin adhesion complexes and thus support cell-shape changes [30], the failure to localize or activate a Rho family GTPase in the absence of EB3 would also explain the defects in myoblast elongation we report here. It has been described that cell fusion occurs preferentially at the tips of elongated myoblasts [10]. We therefore think that successful cell polarization is a prerequisite to the acquisition of fusion competence and thus that the myoblast-fusion effect observed in EB3-depleted cells is a secondary effect, resulting from defects in cytoskeletal organization and cell polarization. It will be a future challenge to analyze EB3's precise role during myogenic differentiation, its effect on Rho/Rac/Cdc42 signaling, and the interplay with EB-interacting proteins that modulate microtubule stability at the cell cortex. Because EB3 is preferentially expressed in the central nervous system [6], insights gained from EB3-dependent processes during myogenesis might contribute to our understanding of neuronal differentiation, as well.

## Figures and Tables

**Table 1:** Summary of Myoblast-Fusion-Efficiency and Microtubule-Dynamics Data

	Control	dEB3	dEB3+EB3	dEB3+EB1	dEB3+EB3-EB1
Fusion efficiency	1.0	0.30 ± 0.07	0.93 ± 0.10	0.35 ± 0.09	0.94 ± 0.15
Growth time at cortex (s)	1.7 ± 0.28 (n = 64)	20.7 ± 6.13 (n = 38)	3.3 ± 0.99 (n = 55)	1.8 ± 0.88 (n = 54)	1.1 ± 0.65 (n = 91)
Percent of microtubules that “overgrow”	21.9%	89.5%	58.2%	38.9%	17.6%
Dwell time at cortex (s)	320 ± 120 (n = 130)	106 ± 22 (n = 116)	319 ± 74 (n = 93)	119 ± 62 (n = 154)	343 ± 69 (n = 132)
Growth rate (μm/s)	0.165 ± 0.065 (n = 94)	0.176 ± 0.083 (n = 98)	0.147 ± 0.042 (n = 38)	0.139 ± 0.057 (n = 40)	0.176 ± 0.057 (n = 52)
Shrinkage rate from cell cortex (μm/s)	0.562 ± 0.150 (n = 50)	0.389 ± 0.171 (n = 76)	0.520 ± 0.130 (n = 40)	0.378 ± 0.141 (n = 61)	0.471 ± 0.138 (n = 51)

## Supplemental Data

Document S1. Experimental Procedures and Six Figures

Movie S1. Microtubule Dynamics at the Cell Cortex in Cells Transfected with Luciferase shRNAs Time-lapse images of C2C12 cells that were transfected with hairpin constructs against luciferase and GFP-tubulin as a reporter 3 days before imaging and differentiated for 2 days. The scale bar represents 2 μm. The relative time is indicated in the lower left corner.

Movie S2. Microtubule Dynamics at the Cell Cortex in Cells Transfected with EB3 shRNAs Time-lapse images of C2C12 cells that were transfected with hairpin constructs against EB3 and GFP-tubulin as a reporter 3 days before imaging and differentiated for 2 days. The scale bar represents 2 μm. The relative time is indicated in the lower left corner.
