# Supplementary material for: EB3 Regulates Microtubule Dynamics at the Cell Cortex and Is Required for Myoblast Elongation and Fusion
Source: Curr Biol. 2007 Aug 7;17(15):1318–25. doi: 10.1016/j.cub.2007.06.058 (PMC1971230; doi:10.1016/j.cub.2007.06.058)
Supplement: Document S1. Experimental Procedures and Six Figures [file mmc1.pdf]

Supplemental Data

EB3 Regulates Microtubule Dynamics at the Cell Cortex and Is Required for Myoblast Elongation and Fusion

Anne Straube and Andreas Merdes

Supplemental Experimental Procedures

RT-PCR Analysis

RNA was isolated from undifferentiated and differentiated C2C12 cells with Trizol Reagent (GIBCO), a chloroform wash, and RNA precipitation with isopropanol. One microgram of total RNA was used for first stand synthesis with random hexamer primer and SuperScript II Reverse Transcriptase (Invitrogen) according to the manufacturer's protocol. One microliter of the resulting cDNA was subsequently used for PCR with specific primers for EB1, EB2, and EB3 that were designed to amplify the complete coding sequence of each gene.

Cloning of Expression and shRNA Constructs

Depletion constructs were based on pSUPERneo.gfp (Oligoengine). The following RNAi target sequences were used: dEB3, GTGAGAA TCTGAGTCGCCA; dEB1, CAGACAAGGTCAAGAAACT; dEB1+EB3, TGGACATGCTCTTCCCTGG; and luciferase, CGTACGCGGAATACT TCGA, which was used as a control. For obtaining the pSuper variants expressing GFP-tubulin, the neomycin restriction cassette was replaced with the tubulin open reading frame (ORF) from pEGFP-tubulin (Clontech) with BsrGI and BamHI. Long and short forms of EB3 cDNA, as well as the EB1 coding region, were amplified from random primed cDNA, obtained from C2C12 cells that were differentiated for 3 days (see above), and subsequently cloned into AgeI and NotI of pEGFP-N1 (Clontech), thereby replacing the eGFP open reading frame (ORF). For the rescue constructs, five silent point mutations were introduced to the EB3 cDNA by a three-step PCR [S1], resulting in the sequence GTGAGAA<u>C</u>CT <u>CTCA</u>CGCCA. Single point mutations H29Q and Y32L were introduced to EB3 with the same strategy. The double FLAG tag (DYKDADLDKDDDDK) was introduced in two parts by PCR to the upstream and downstream parts of EB1 and EB3 and ligated with a BglII site within the tag. This site was also used for domain swapping at in the chimeric constructs. Domain swapping at position 53 was done with a SapI site that is conserved in the nucleotide sequences of EB1 and EB3. For C-terminal deletion in EB3, a stop codon followed by a NotI site was introduced at the respective sites by PCR.

Cell Culture and Transfection Experiments

Mouse myoblasts C2C12 were cultured in DMEM (Invitrogen) supplemented with 10% fetal bovine serum (FBS) (Perbio), 2 mM L-glutamine, and antibiotics. For shRNA depletion during differentiation, cells were transfected with Lipofectamine Plus (Invitrogen) and shifted to differentiation medium (Dulbecco's modified Eagle's medium [DMEM] + 0.1% FBS + 2 mM L-glutamine + 5 μg/ml insulin + 5 μg/ml transferrin + antibiotics) when cells had grown to confluency. The differentiation medium was exchanged daily, and analysis of shRNA depleted cells was consistently performed 72–76 hr after transfection. To comply with that, cell density at the time of transfection was adjusted to the desired differentiation time. Because cell density is crucial for progression of myoblast fusion, caspase inhibitor Z-VAD-FMK (Calbiochem) was added after transfection to prevent reduction in cell density due to apoptosis. Skeletal myoblasts of *H-2K*^{b}-tsA58 transgenic mice [S2] were grown in DMEM (Invitrogen) supplemented with 20% FBS (Perbio), 2% chicken embryo extract (SLI), 20 μg/ml interferon-γ (Sigma), and antibiotics at 33°C and 6% CO_2. For differentiation, cells were incubated in DMEM supplemented with 2% FBS, 0.2% chicken embryo extract, and antibiotics at 39°C and 5% CO_2.

Live-Cell Imaging and Image Analysis

Cells grown on acid-washed coverslips were mounted in a closed chamber on a heated stage insert (ALA Scientific Instruments) in medium supplemented with 25 mM HEPES. Imaging was done with a Deltavision system. Acquisition and image analysis were performed with Softworx (Applied Precision). Images were exported to Adobe Photoshop and Corel Draw for presentation. Statistical analysis was performed with Origin 6.0 (Microcal). The statistical significance of the observed differences was evaluated with ANOVA tests, and in all plots, standard deviation (SD) is indicated.

Immunofluorescence Microscopy

Cells were fixed either in methanol at −20°C overnight or in 4% paraformaldehyde (EMS) in phosphate buffered saline (PBS) in cytoskeletal buffer (10 mM PIPES, 100 mM NaCl, 300 mM sucrose, 3 mM $MgCl_2$ [pH 6.8]) for 20 min, and then in 50 mM NH_4Cl in PBS for 5 min. Cells were permeabilized in 0.2% Triton X-100 in PBS and stained for immunofluorescence according to standard protocols. The following antibodies were used: anti-EB1 mAB, anti-EB3 mAB (Transduction Labs), anti-EB3 rabbit and anti-EB2 rat antiserum [S3], anti-tubulin mAb clone DM1A (Sigma), anti-detyrosinated tubulin rabbit antiserum [S4], anti-embryonic myosin (DSHB, University of Iowa), anti-PCM-1 [S5], anti-vinculin (Sigma), anti-γtubulin (Sigma), anti-myogenin (Santa Cruz), and donkey anti-rabbit or anti-mouse IgG, conjugated with Alexa 488, Alexa 594, or Alexa 647 (Molecular Probes). DNA was stained with 0.5 μg/ml DAPI (Sigma). Coverslips were mounted with Vectashield (VectorLabs).

Cell Sorting and Western Analysis

Cells were detached from culture dishes with trypsin-ethylenediamine tetraacetic acid (EDTA). For cell sorting, cells were transferred to PBS + 10% FBS (1% for differentiated cells), and GFP-expressing cells were separated with a DakoCytomation MoFlo MLS flow cytometer. Cells were extracted in protein sample buffer and boiled for 5 min. Amounts of extracts equal to 15,000 or 20,000 GFP-expressing cells were loaded onto 10% or 12.5% sodium dodecyl sulfate-polyacrylamide gel electrophoresis (SDS-PAGE) gels. Primary antibodies were used as for immunofluorescence and detected with goat anti-mouse pAb HRP (Promega), donkey anti-rabbit IgG pAb horseradish peroxidase (HRP), and anti-rat pAb HRP (both Amersham), and ECL (Amersham) or SuperSignal West Dura Extended substrate (Pierce) followed.

Cell-Spreading Experiments

Coverslips were coated with 10 μg/ml fibronectin, collagen, laminin, or poly-D-lysine (all from Sigma) for 24 hr at room temperature (RT), washed with water, and allowed to dry. GFP-expressing C2C12 cells were enriched by FACS sorting (see above) 1 day after transfection with pSuper constructs and seeded at high density to allow shifting to differentiation conditions the day after sorting. Three days after transfection, cells were trypsinized, and an equal amount of cells was diluted in 1 ml DMEM + 1% FBS and allowed to settle on coverslips coated with different substrates for 70 min.

3D-Structure Analysis

Structural data for EB1 and EB3 were obtained from protein data bank at http://www.rcsb.org/pdb under accession numbers 1PA7 and 1WYO, respectively. To compare EB1 and EB3 structures, the data file for EB3 was modified with SYBYL 6.9.1., thereby removing the first seven amino acids that were a cloning artifact and the last 12 amino acids, which were not included in the EB1 structure and appeared to be highly flexible. Both structures were superimposed and surface representation calculated with SYBYL. Other structure representations were done with Protein Explorer 2.78 (http://www.proteinexplorer.org) on the modified file for 1WYO.

Supplemental References

S1. Picard, V., Ersdal-Badju, E., Lu, A., and Bock, S.C. (1994). A rapid and efficient one-tube PCR-based mutagenesis technique using *Pfu* DNA polymerase. Nucleic Acids Res. *22*, 2587–2591.

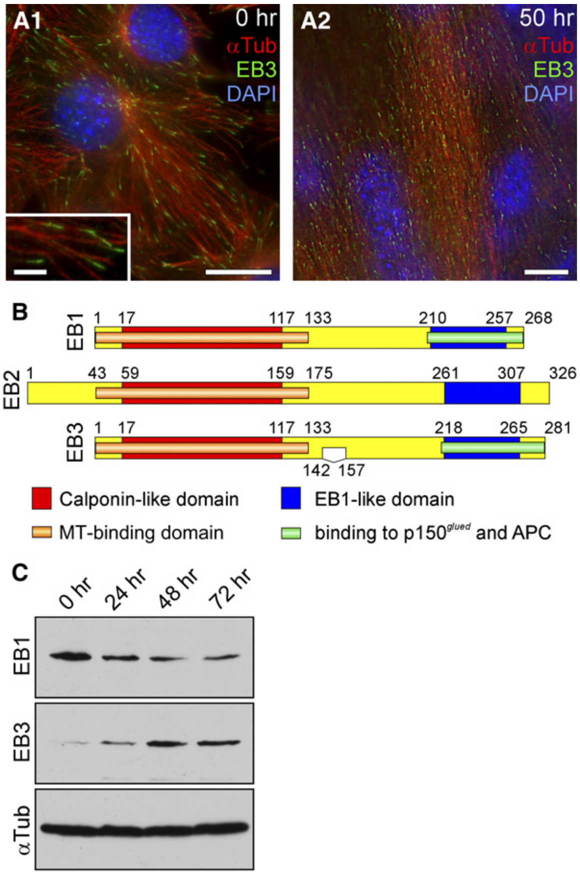

Figure S1. EB Proteins

(A) Immunofluorescence of undifferentiated (A1) and differentiated (A2) C2C12 cells that were stained for EB3 (green), α-tubulin (red), and DAPI (blue). The inset shows microtubule tips at higher magnification. The scale bar represents 10 μm, and 2 μm in inset.

(B) Domain structure of proteins of the EB family in mouse. All three contain an N-terminal Calponin-like domain (>70% identity) that confers microtubule binding and a C-terminal domain (>50% identity) that is involved in dimerization [S6] and binding to dynactin and APC [S7]. The two alternatively spliced forms of EB3 [S8] are indicated with the gap between amino acids 142 and 157.

(C) Western blots of total cell lysates of *H-2K*^{b}-tsA58 myoblasts that were cultured under differentiation conditions for the times indicated and probed with antibodies against EB1, EB3, and α-tubulin.

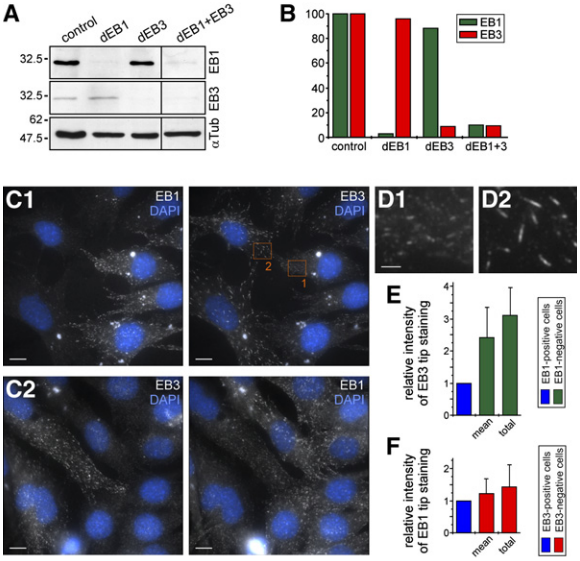

Figure S2. Depletion of EB1 and EB3 with shRNA Constructs

(A) Western blots of total cell lysates of GFP-expressing C2C12 myoblasts 3 days after transfection with hairpin RNA constructs and subsequent FACS sorting. Blots were probed with antibodies against EB1, EB3, and α-tubulin. Positions of molecular weight markers are indicated on the left.

(B) Quantification of EB1 (green) and EB3 (red) protein levels in cells expressing the shRNA constructs indicated above.

(C) Immunostaining of EB1 and EB3 in EB1- (C1) or EB3- (C2) depletion experiments. Images show depleted and undepleted cells. Scale bars represent 10 μm.

(D) EB3 staining in an EB1 positive (D1) and an EB1 negative (D2) cell at higher magnification. Images correspond to the orange boxes in C1. Note that the intensity as well as the size of the EB3 signal at microtubule tips is increased in EB1-depleted cells. The scale bar represents 2 μm.

(E) Quantification of EB3 signals at microtubule tips in EB1-depletion experiments. Mean intensity as well as total intensity per microtubule tip is shown relative to levels in EB1-positive cells in the same image. Data show mean values from six images, and error bars represent the SD.

(F) Quantification of EB1 signals at microtubule tips in EB3-depletion experiments. Mean intensity as well as total intensity per microtubule tip is shown relative to levels in EB3-positive cells in the same image. Data show mean values from six images, and error bars represent the SD.

S2. Morgan, J.E., Beauchamp, J.R., Pagel, C.N., Peckham, M., Ataliotis, P., Jat, P.S., Noble, M.D., Farmer, K., and Partridge, T.A. (1994). Myogenic cell lines derived from transgenic mice carrying a thermolabile T antigen: A model system for the derivation of tissue-specific and mutation-specific cell lines. Dev. Biol. *162*, 486–498.

S3. Komarova, Y., Lansbergen, G., Galjart, N., Grosveld, F., Borisy, G.G., and Akhmanova, A. (2005). EB1 and EB3 control CLIP dissociation from the ends of growing microtubules. Mol. Biol. Cell *16*, 5334–5345.

S4. Gundersen, G.G., Khawaja, S., and Bulinski, J.C. (1989). Generation of a stable, posttranslationally modified microtubule array is an early event in myogenic differentiation. J. Cell Biol. *109*, 2275–2288.

S5. Dammermann, A., and Merdes, A. (2002). Assembly of centrosomal proteins and microtubule organization depends on PCM-1. J. Cell Biol. *159*, 255–266.

S6. Honnappa, S., John, C.M., Kostrewa, D., Winkler, F.K., and Steinmetz, M.O. (2005). Structural insights into the EB1-APC interaction. EMBO J. *24*, 261–269.

S7. Bu, W., and Su, L.K. (2003). Characterization of functional domains of human EB1 family proteins. J. Biol. Chem. *278*, 49721–49731.

S8. Su, L.K., and Qi, Y. (2001). Characterization of human MAPRE genes and their proteins. Genomics *71*, 142–149.

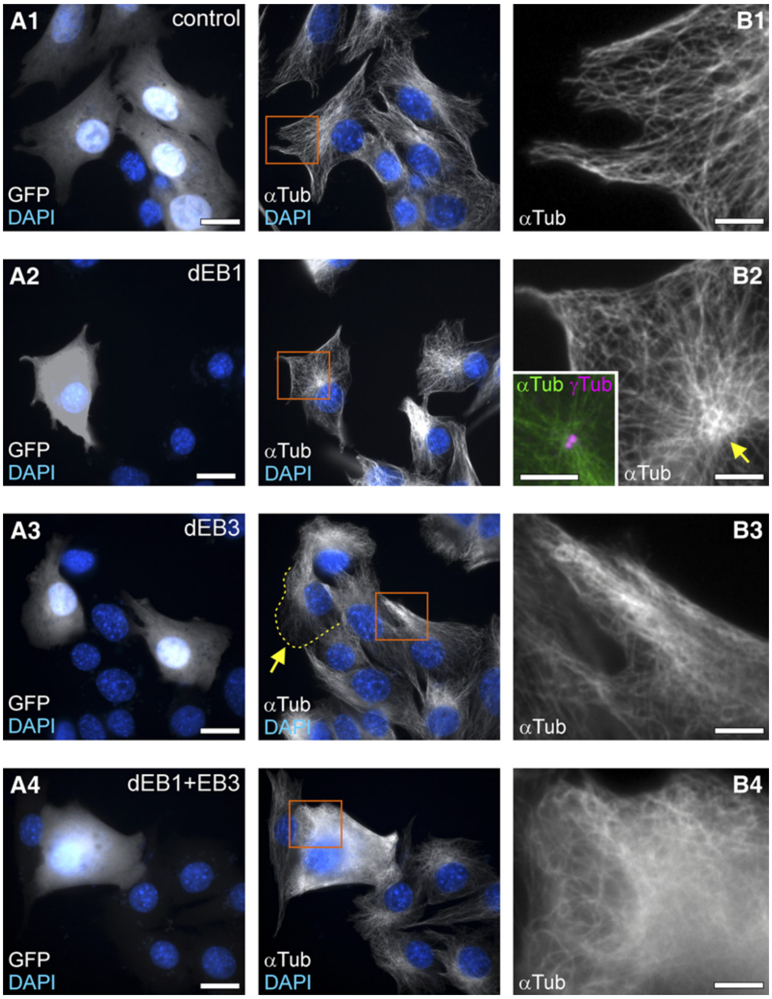

Figure S3. Depletion of EB1, EB3, and EB1+EB3 in Undifferentiated Myoblasts

Microtubule organization in C2C12 cells expressing shRNA constructs against luciferase (control), EB1, EB3, and EB1+EB3 simultaneously and coexpressing GFP as a reporter. The left panels show GFP (white), and the center and right panels show tubulin immunofluorescence (white). Nuclei are indicated in blue in both panels. (B1) to (B4) show the boxed areas in (A) at higher magnifications. Note that cells negative for GFP might nevertheless express the hairpin construct. The centrosomal focus of microtubules is highlighted by a yellow arrow in (B2), and the inset shows GFP-tubulin (green) and γ-tubulin immunofluorescence (magenta). Scale bars represent 20 μm in (A) and 5 μm in (B).

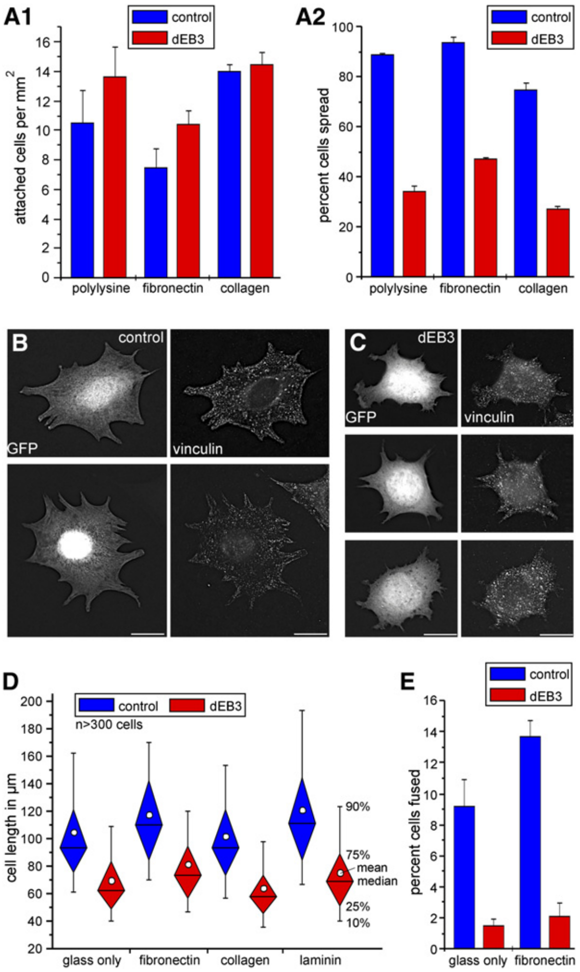

Figure S4. Myoblast Adhesion, Spreading, Elongation, and Fusion on Different Substrates

(A) Cell attachment and spreading efficiency 70 min after seeding control and EB3-depleted cells on polylysine-, fibronectin-, and collagen-coated coverslips. Columns represent the number of attached cells per mm^2 (A1) or the percentage of spread cells (A2) averaged from two experiments, and error bars indicate the SD.

(B and C) Representative images of cells spread on fibronectin. GFP highlights cells that are transfected with control (B) or EB3-specific shRNAs (C), and focal adhesions are stained with vinculin antibodies. Scale bars represent 20 µm.

(D) Cell-length distribution in differentiated myoblasts after 40 hr, cultured on either glass coverslips only or coverslips coated with fibronectin, collagen, or laminin. Diamond-shaped boxes represent 25% and 75% at the tips and the median in the middle, and whiskers 10% and 90%. Mean values are indicated by a white dot.

(E) Myoblast-fusion efficiency was determined on cells cultured on fibronectin versus glass only, 3 days after transfection with hairpin RNA constructs and after 54 hr under differentiation conditions. Columns represent the percentage of cells containing two or more nuclei, averaged from two experiments, and error bars indicate the SD.

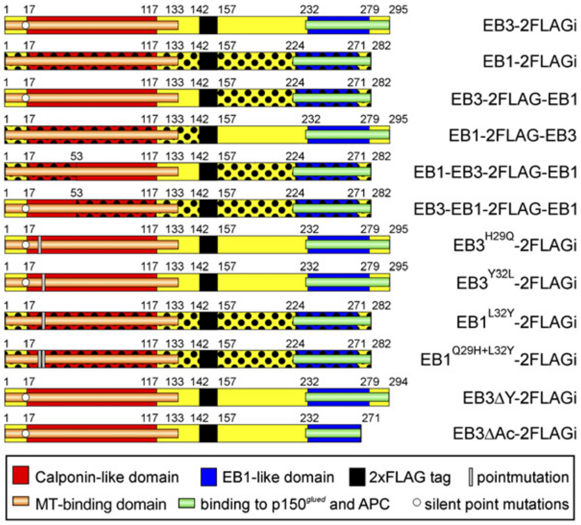

Figure S5. Rescue Constructs

Schematic representation of constructs used to rescue fusion defects of EB3 depletion. All constructs contain an internal double FLAG tag (abbreviated as 2FLAGi) that is inserted just before the alternative splice site in EB3 and at the respective site in EB1. The polka-dot pattern highlights sequence stretches that were derived from EB1.

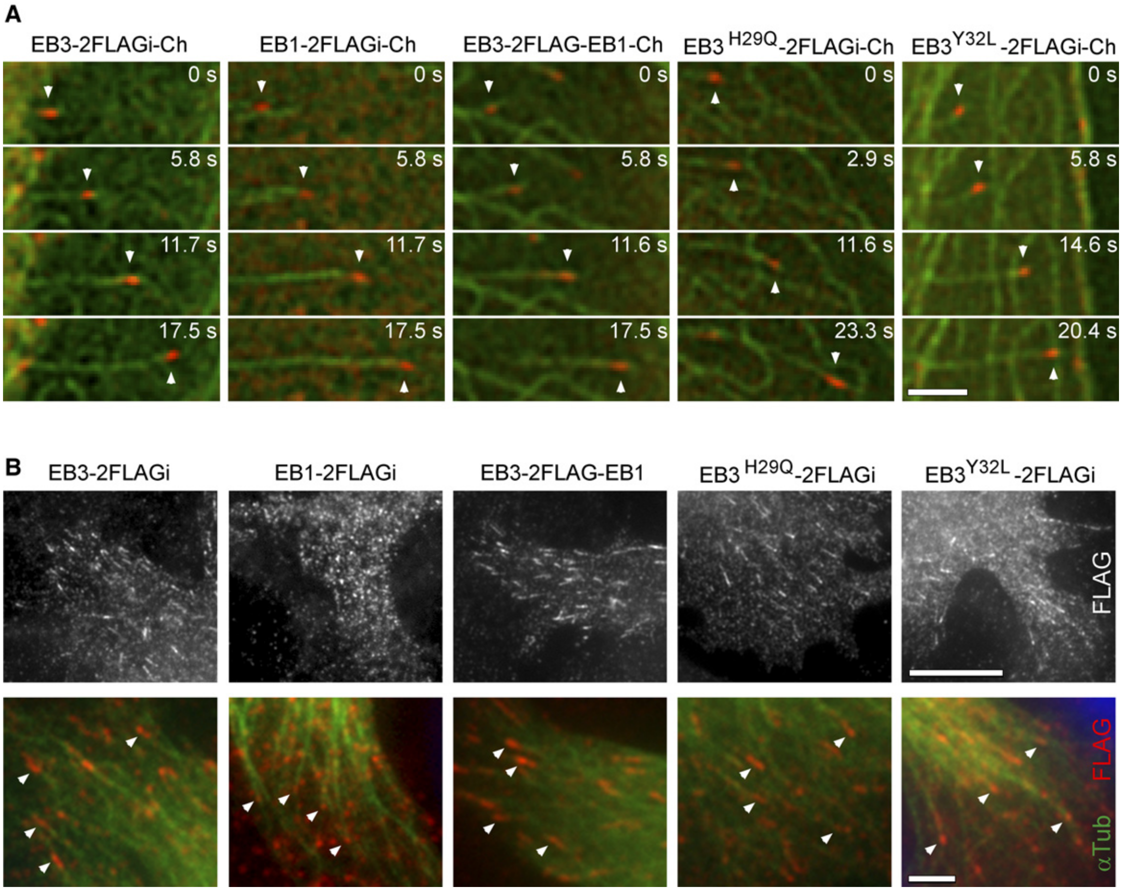

Figure S6. Tip Tracking and Localization of Rescue Constructs

(A) Stills from time-lapse movies of GFP-tubulin and various rescue constructs fused to mCherry, demonstrating plus-end tracking ability for all constructs shown. The relative time is indicated in the upper-right corner, and arrowheads highlight the microtubule plus end in each frame. The scale bar represents 2 μm.

(B) Immunolocalization of rescue constructs in undifferentiated C2C12 cells with FLAG M2 antibodies. Colocalization with microtubules is shown at higher magnification in the lower row. Arrowheads highlight signals at microtubule tips. Scale bars represent 10 μm in the upper row and 2 μm in the lower row.
